# A novel disulfidptosis-associated expression pattern in breast cancer based on machine learning

**DOI:** 10.3389/fgene.2023.1193944

**Published:** 2023-06-29

**Authors:** Zhitang Wang, Xianqiang Du, Weibin Lian, Jialin Chen, Chengye Hong, Liangqiang Li, Debo Chen

**Affiliations:** Department of Breast, The First Hospital of Quanzhou Affiliated to Fujian Medical University, Quanzhou, China

**Keywords:** breast cancer, disulfidptosis, prognostic signature, tumor microenvironment, expression pattern

## Abstract

**Background:** Breast cancer (BC), the leading cause of cancer-related deaths among women, remains a serious threat to human health worldwide. The biological function and prognostic value of disulfidptosis as a novel strategy for BC treatment via induction of cell death remain unknown.

**Methods:** Gene mutations and copy number variations (CNVs) in 10 disulfidptosis genes were evaluated. Differential expression, prognostic, and univariate Cox analyses were then performed for 10 genes, and BC-specific disulfidptosis-related genes (DRGs) were screened. Unsupervised consensus clustering was used to identify different expression clusters. In addition, we screened the differentially expressed genes (DEGs) among different expression clusters and identified hub genes. Moreover, the expression level of DEGs was detected by RT-qPCR in cellular level. Finally, we used the least absolute shrinkage and selection operator (LASSO) regression algorithm to establish a prognostic feature based on DEGs, and verified the accuracy and sensitivity of its prediction through prognostic analysis and subject operating characteristic curve analysis. The correlation of the signature with the tumor immune microenvironment and tumor stemness was analyzed.

**Results:** Disulfidptosis genes showed significant CNVs. Two clusters were identified based on three DRGs (DNUFS1, LRPPRC, SLC7A11). Cluster A was found to be associated with better survival outcomes(*p* < 0.05) and higher levels of immune cell infiltration(*p* < 0.05). A prognostic signature of four disulfidptosis-related DEGs (KIF21A, APOD, ALOX15B, ELOVL2) was developed by LASSO regression analysis. The signature showed a good prediction ability. In addition, the prognostic signature in this study were strongly related to the tumor microenvironment (TME), tumor immune cell infiltration, tumor mutation burden (TMB), tumor stemness, and drug sensitivity.

**Conclusion:** The prognostic signature we constructed based on disulfidptosis-DEGs is a good predictor of prognosis in patients with BC. This prognostic signature is closely related to TME, and its potential correlation provides clues for further studies.

## 1 Introduction

Breast cancer (BC), the most frequently diagnosed malignancy in women, is a highly heterogeneous disease that accounted for 30% of female malignancies in 2020. This malignancy poses a great threat to women’s health, due to its extremely high recurrence and mortality rates (Siegel et al., 2019; Sung et al., 2021). At present, treatment strategies for BC mainly include surgery, radiotherapy, chemotherapy, hormone therapy, targeted therapy, and immunotherapy. Despite these, however, the mortality rate for BC remains very high (Wang et al., 2021). Therefore, it is imperative to explore new therapeutic targets and reliable prognostic models in order to achieve optimal BC clinical outcomes.

Disulfidptosis is a new type of programmed cell death that has been found to be independent of apoptosis, iron death, necrotic apoptosis, and copper death (Vanden Berghe et al., 2014; Liu et al., 2023). Disulfidptosis is a rapid cell death mechanism caused by disulfide stress resulting from the accumulation of excess cysteine in cells, which usually occurs during glucose starvation (Liu et al., 2023). In glucose-deficient cancer cells expressing high levels of SLC7A11, a large accumulation of disulfide molecules leads to abnormal disulfide formation in the actin cytoskeleton, interfering with the organization of tissues and ultimately leading to the breakdown of the actine network and eventual cell death (Liu et al., 2020). We identified several genes involved in disulfidptosis that may provide novel strategies for predicting outcomes in patients with BC.

This study systematically studied the genomic characteristics of BC-specific disulfidptosis-related genes (DRGs). Based on DRGs, two disulfidptosis expression patterns were determined by unsupervised consensus clustering. The differences in prognosis, clinicopathological factors, and immune features between the two clusters were elucidated. In addition, the prognostic signature based on differentially expressed genes (DEGs) between the two disulfidptosis subtypes has been established to quantify disulfidptosis-related characteristics, high risk score predicted poor prognosis and higher TMB in BC patients. We then analyzed tumor microenvironment (TME) evaluation scores, tumor mutation burden (TMB) associations, RNA based stemness scores (RNAss) associations, and differences in chemotherapy sensitivity in the high-low risk group. These results suggest that disulfidptosis related genes play an important role in BC, which helps us to evaluate the prognosis of patients with BC and their response to chemotherapy and immunotherapy, and these genes may be potential synergistic targets to improve the therapeutic efficacy of BC.

## 2 Methods

### 2.1 Public data acquisition and preprocessing

Disulfidptosis-related gene lists were acquired from recently published literature (Liu et al., 2023). The gene expression data, corresponding survival information, copy number variations (CNVs), and somatic mutation data of patients with BC were obtained from The Cancer Genome Atlas (TCGA) database. Bulk RNA expression matrices were calibrated to the TPM format for subsequent analysis, and the GSE86166 and TCGA-BRCA bulk RNA expression matrices were integrated to form a complete queue. The data were then randomly divided at a ratio of 1:1, into training and test cohorts for subsequent analyses.

The “maftools” R package (version 4.2.2) was used to characterize DRGs and tumor mutation burden (TMB). The “ggpubr” R package was used to analyze the correlation between risk score and TMB, and the boxplot and correlation graph were used to visualize the results. Based on the CNV data, we analyzed the frequency of CNVs in DRGs and used the “RCircos” R package to locate CNVs on the 22 somatic human chromosomes, as well as the X/Y sex chromosomes.

### 2.2 Screening of BC-specific disulfidptosis-related genes

We investigated the differences in the expression levels of DRGs between tumor and normal samples. Statistical significance was considered to be *p* < 0.05. Univariate Cox regression and Kaplan–Meier (KM) analyses were used to screen for BC-specific DRGs. The “limma” and “reshape2” R packages were used to screen DRGs. The KM survival analysis and univariate Cox analysis based on above genes were performed using the R packages “survival” and “survminer.” Venn diagrams were constructed using the R packages “ggplot2” and “VennDiagram.”

### 2.3 Unsupervised clutering for disulfidptosis-related genes

A consensus clustering algorithm based on the R package “ConsensuClusterPlus” with 1000 permutations was used to calculate the number of disulfidptosis clusters in the overall cohorts. Principal component analysis (PCA) was conducted to verify the expression patterns using the R packages “limma” and “ggplot2.”

### 2.4 Functional enrichment analysis

Gene Ontology (GO) and Kyoto Encyclopedia of Genes and Genomes (KEGG) pathway enrichment analyses were performed for patients between high- and low-risk groups using the “clusterProfiler” R package. Statistical significance was set at *p* < 0.05 for GO and KEGG pathways.

### 2.5 Analysis of correlation with immune infiltration

Based on the LM22 gene set on the CIBERSORT website, the CIBERSORT algorithm was used to estimate the total immune infiltration of high- and low-risk groups, as well as DRGs.

### 2.6 Screening of hub disulfidptosis-related DEGs

Gene expression between clusters was compared by “limma” R package, and the differentially expressed genes were obtained according to | FC | > 1, *p* < 0.05. These genes were included in univariate Cox analysis to obtain genes with important value. Least absolute shrinkage and selection operator (LASSO) Cox regression was used for 10-fold cross-validation of overall survival (OS), and genes related to disulfidptosis were screened. The “glmnet” R package was used to identify genetic signatures containing biomarkers that were the most helpful for prognosis, and risk scores were calculated for each sample in all datasets based on these signatures. The risk score was calculated using the following formula:

$$Risk\;score=\left(KIF21A*0.218\right)+\left(APOD*-0.058\right)+\left(ALOX15B*-0.071\right)+\left(ELOVL2*-0.087\right)$$

To assess the predictive ability of disulfidptosis-related differentially expressed genes (DEGs), time-dependent receiver operating characteristic (ROC) at 3 years, 5 years, and 10 years of survival were analyzed in training and test data sets using the “timeROC” R package. For survival analysis, the optimal cut-off value of risk score was analyzed using the “Survival” R package, and the samples were divided into a high-risk and low-risk group. Kaplan–Meier analysis was used to investigate the prognostic significance of disulfidptosis-related DEGs. In addition, a prognostic nomogram was established based on the TCGA-BC dataset. Time-dependent calibration curves were plotted to predict the accuracy of the nomogram.

### 2.7 Cell culturing

The cell lines used in the study included human normal breast cell line MCF-10A and human breast cancer cell line MDA-MB-231were purchased from Procell (Wuhan, China). Cells were cultured in DMEM medium supplemented with 10% FBS (Gibco, United States) and antibiotics (Penicillin 100 U/mL, Streptomycin 100 mg/mL) (Gibco, United States). Cells were cultured at 37°C with 5% CO2.

### 2.8 RNA extraction and quantitative real-time PCR (qRT-PCR)

RNA was isolated using TRIzol reagent (Invitrogen, Thermo Fisher Scientific, Waltham, MA, United States), and reverse transcription was performed using the PrimeScriptTM RT Reagent Kit (Takara; Takara Bio, Shiga, Japan). SYBR Green PCR Master Mix (Takara) was used for qRT-PCR on a StepOnePlus System (Applied Biosystems, Thermo Fisher Scientific). Fold-changes in gene expression were determined using the 2^−ΔΔCT^ method, using GAPDH for normalization. The primers used in this study are listed in Supplementary Table S1.

### 2.9 Statistical analysis

The Wilcoxon rank-sum test was used to compare differences between the two groups. The K–W test was performed to compare three or more groups. Kaplan–Meier analysis was used to evaluate survival differences between the low- and high-risk- groups. All statistical analyses were done using R version 4.2.2 with *p* < 0.05 indicating statistical significance.

## 3 Results

### 3.1 Genetic alterations analysis and screening of disulfidptosis-related genes in BC

We identified 10 genes (*NCKAP1*, *LRPPRC*, *NDUFS1*, *GYS1*, *SLC3A2*, *RPN1*, *SLC7A11*, *OXSM*, *NDUFA11*, and *NUBPL*) that were closely related to disulfidptosis. We first determined the somatic mutation levels, CNVs, gene expression levels, and prognostic values of DRGs in BC samples.

**FIGURE 1:**
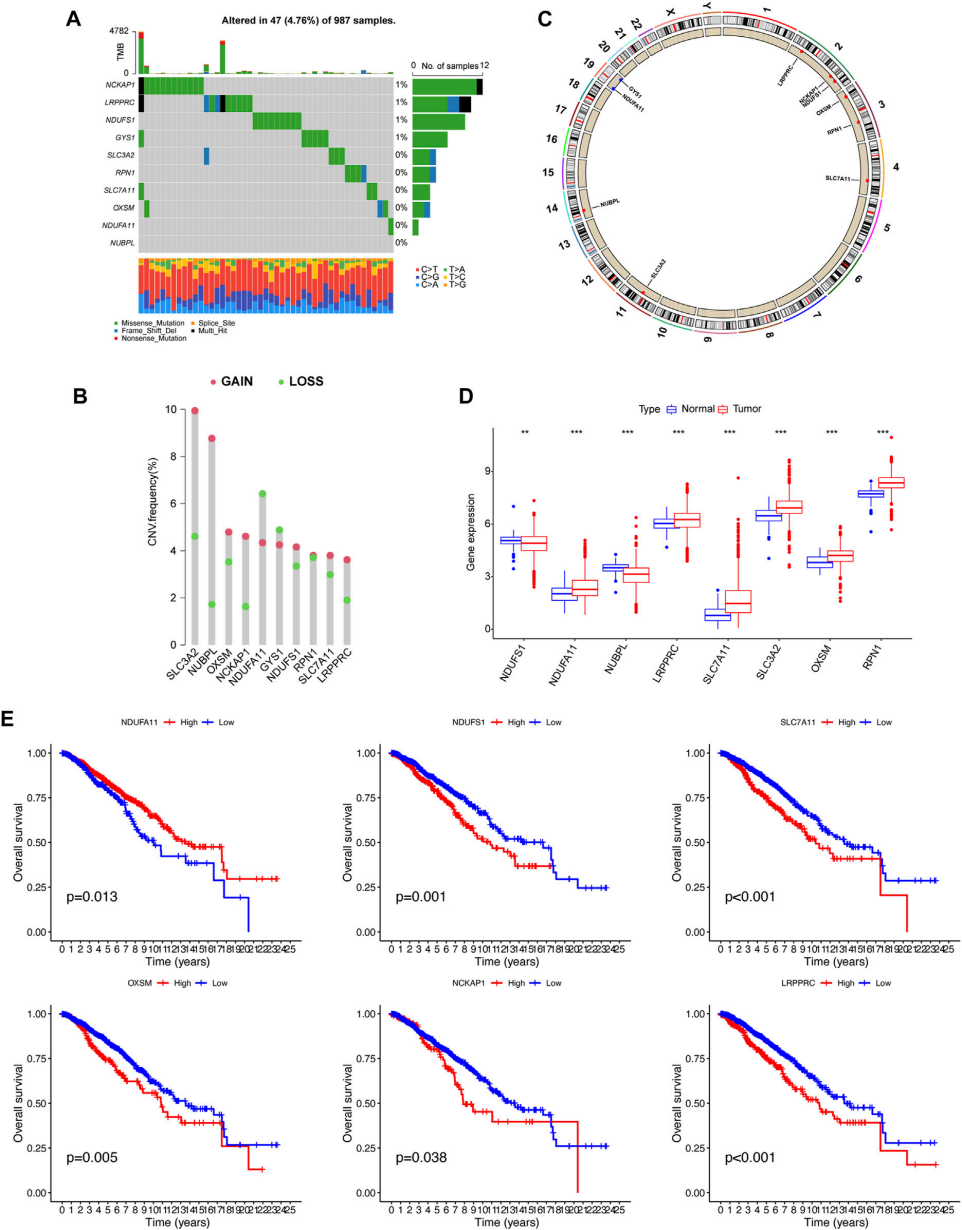
Gene mutational, copy number variations (CNV), differentially expressed, and survival analysis of disulfidptosis-related genes. **(A)** Waterfall plot showing the gene mutational frequency and types of genetic mutations. **(B, C)** Bar chart and circus show the CNV frequency and the position of the disulfidptosis-related genes on the chromosomes. **(D)** Gene expression analysis between normal and breast cancer samples. **(E)** K–M survival analysis between high and low expression of genes. **p < 0.01, ***p < 0.001.

Somatic mutations were not widespread in these genes (Figure 1A). Somatic mutations in the DRGs were present in 47 of the 987 samples, a frequency of 4.76%. Among these, the mutation frequencies of *NCKAP1*, *LRPPRC*, *NDUFS1*, and *GYS1* were the highest. By investigating the frequency of the CNVs, we noticed that DRGs had widespread alterations in CNVs and that most genes had a gain status that was higher than the loss status. The primary genes showing CNV amplification were *SLC3A2* and *NUBPL*. By contrast, *NDUFA11* had the highest number of CNV deletions (Figure 1B). The positions of these 10 genes on the chromosome are shown in Figure 1C. We then analyzed the expression levels of these 10 genes in cancers and their adjacent normal tissues. *NDUFA11*, *LRPPRC*, *SLC7A11*, *SLC3A2*, *OXSM*, and *RPN1* showed higher expression levels in cancer tissues, whereas *NDUFS1* and *NUBPL* were expressed at lower levels (*p* < 0.01). The expression of *NCKAP1* and *GYS1* was not significantly different between cancer and adjacent normal tissues (Figure 1D). OS analysis showed that the group with high expression of *NDUFA11* and the group with low expression of *NDUFS1*, *SLC7A11*, *OXSM*, *NCKAP1*, and *LRPPRC* had better prognoses (*p* < 0.05; Figure 1E). There were no significant differences in OS between the *NUBPL*, *RPN1*, and *SLC3A2* expression groups.

### 3.2 Identification of BC-specific DRGs and distinct expression patterns

**FIGURE 2:**
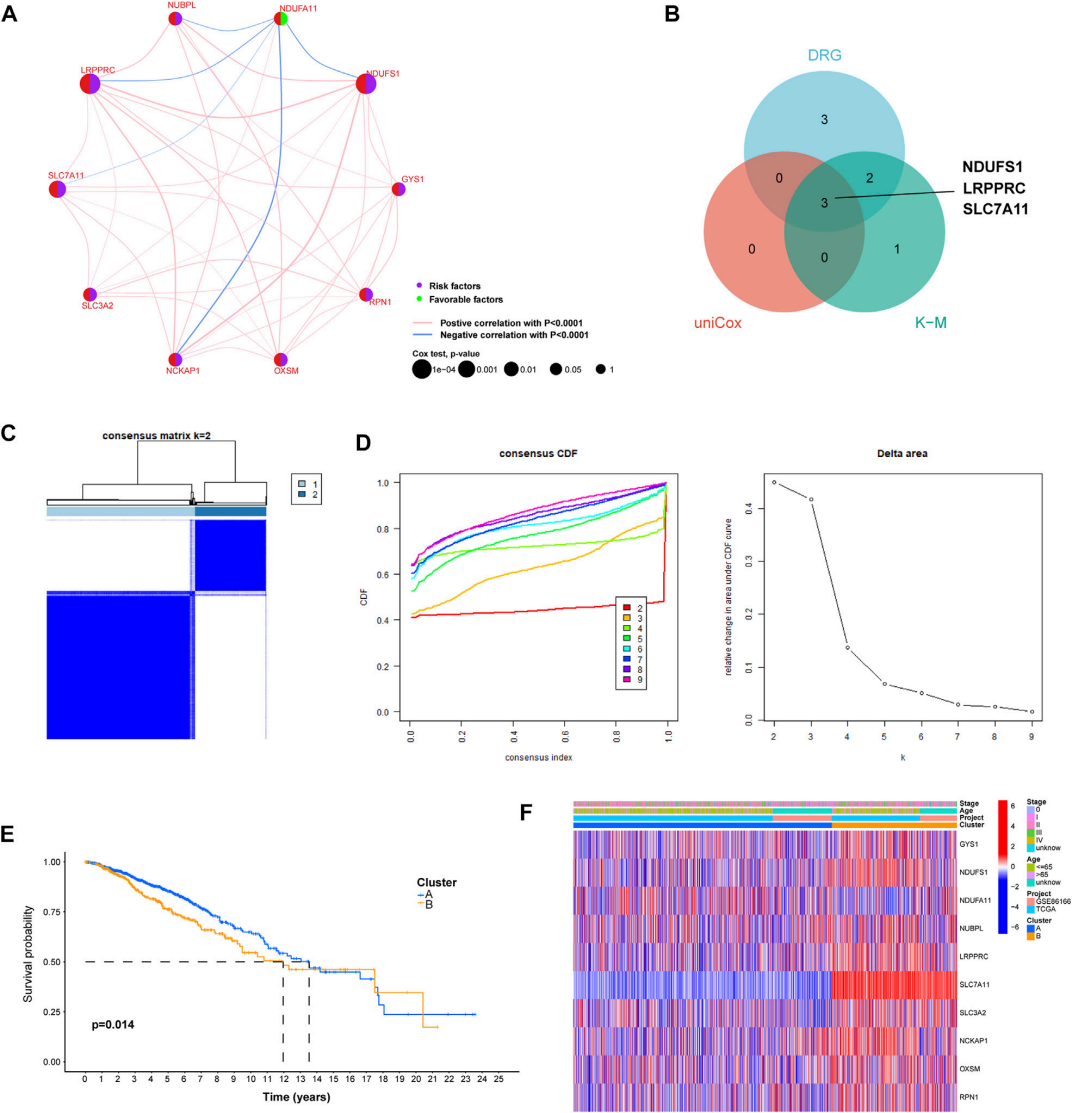
The construction of distinct disulfidptosis-related expression patterns. **(A)** Univariate Cox regression and correlation analysis between disulfidptosis-related genes. **(B)** Venn plot showing the shared genes according to the results of differentially expressed analysis, univariate Cox regression analysis, and K–M survival analysis. **(C)** The consensus clustering matrix (*k* = 2) was used to stratify Breast cancer (BC) patients into two clusters. **(D)** Consensus clustering model with cumulative distribution function (CDF) by k from 2-9. **(E)** K-M survival analysis between cluster A and **(B) (F)** The heat map shows differences in clinicopathological factors in each distinct cluster.

Univariate Cox regression analysis identified three primary genetic risk factors: *LRPPRC*, *NDUFS1*and *SLC7A11* (*p* < 0.01; Figure 2A). Three BC-specific DRGs were identified by intersections of eight DRGs, six prognostic DRGs, and three risk factors from the univariate cox regression analysis. These were the genes *NDUFS1*, *LRPPRC* and *SLC7A11* (Figure 2B). Based on these genes, unsupervised consensus clustering of the overall cohort was performed and patients with BC in the overall cohort were categorized into clusters A and B (Figures 2C, D). PCA showed that BC samples could be distinguished according to distinct expression patterns, and our KM survival curve showed that the median OS of cluster A was better than that of cluster B (Figure 2E).

### 3.3 Correlation between expression patterns and BC molecular subtype

We constructed a heat map that showed the differences in the clinical factors between clusters A and B (Figure 2F). In order to further explore the relationship between breast cancer molecular subtypes and the expression pattern we identified, we drew the Sankey diagram and KM survival curve. The results showed that in cluster A, patients with luminal A, luminal B, HER2 and Basel subtypes account for 60.4%, 17.4%, 8.8% and 13.4%, respectively. In cluster B, luminal A, luminal B, HER2 and Basel subtypes accounted for 39.8%, 26.0%, 5.8% and 28.4%, respectively (Supplementary Figure S1A). The results indicated that the proportion of patients with Luminal A subtype in cluster A is significantly higher than that in cluster B, and the proportion of patients with Basel subtype in cluster B is significantly higher than that in cluster A. The results of KM survival analysis showed that there was a significant difference in the prognosis of patients in the cluster A and B of luminal subtype, but no difference was found in HER2 and Basel subtypes. (Supplementary Figure S1B).

### 3.4 Analysis immune infiltrate level analysis and functional enrichment analysis between two clusters

**FIGURE 3:**
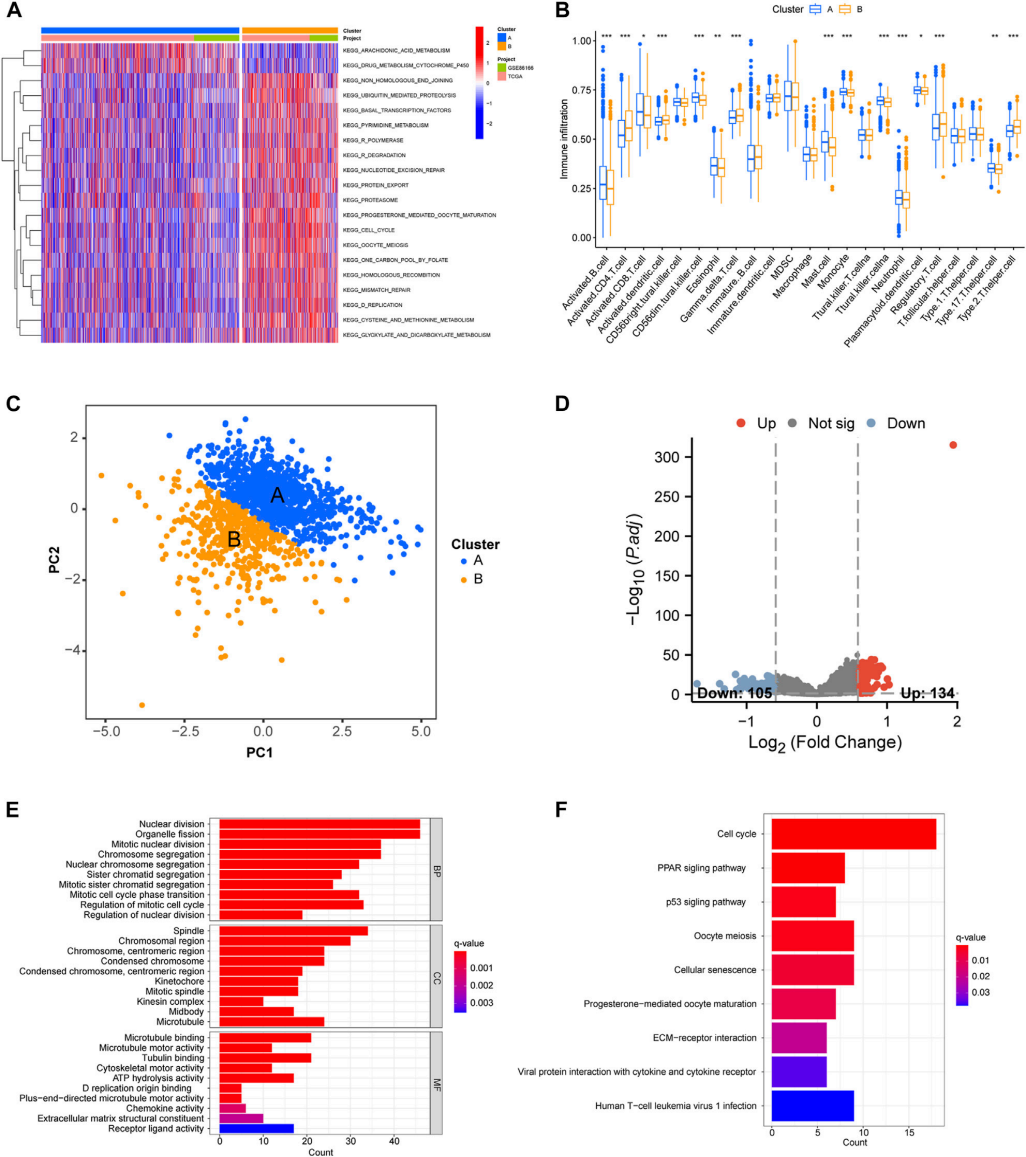
ssGSEA and immune infiltration analysis in distinct cluster and functional enrichment analysis of disulfidptosis. **(A)** Heat map plot showing our ssGSEA analysis of clusters **(A, B)**. **(B)** Box plot showing the differences between clusters **(A, B)**. **(C)** Principal Component Analysis (PCA) based on the two clusters. **(D)** The differentially expressed genes between cluster **(A, B)**. **(E, F)** GO and KEGG analysis of molecular subtype-related DEGs. *p < 0.05, **p < 0.01, ***p < 0.001.

GSVA functional enrichment analysis indicated that cluster A was mainly enriched in mutations pertaining to arachidonic acid and drug metabolism pathways. Cluster B was mainly enriched in tumor-related pathways (e.g., DNA replication and cell cycle) and metabolic pathways (e.g., primary bile acid biosynthesis, pyrimidine metabolism, cysteine and methionine metabolism, and glyoxylate and dicarboxylate metabolism; Figure 3A). As shown in Figure 3B, the extent of immune cell infiltration differed distinctly between clusters A and B. CD56^bright^ natural killer cells, immature B cells, immature dendritic cells, MDSC, macrophages, natural killer T cells, follicular helper T cells and Type 1 helper T cells were observed. The infiltration of immune cells was higher in cluster A than in cluster B. PCA analysis showed that cluster A and B could better distinguish patients into different group. Therefore, we further explored the difference between the two clusters (Figure 3C). 239 disulfidptosis-DEGs were identified between cluster A and B (Figure 3D). GO and KEGG enrichment analyses of disulfidptosis-DEGs showed that these genes were mainly enriched in cell division-related pathways (e.g., nuclear division, mitotic nuclear division, and chromosome segregation; Figure 3E). The results of the KEGG analysis showed that disulfidptosis-DEGs were significantly enriched in cancer-related pathways (e.g., cell cycle, p53 signaling pathway, and ECM-receptor interaction; Figure 3F).

### 3.5 Construction of prognostic signature

**FIGURE 4:**
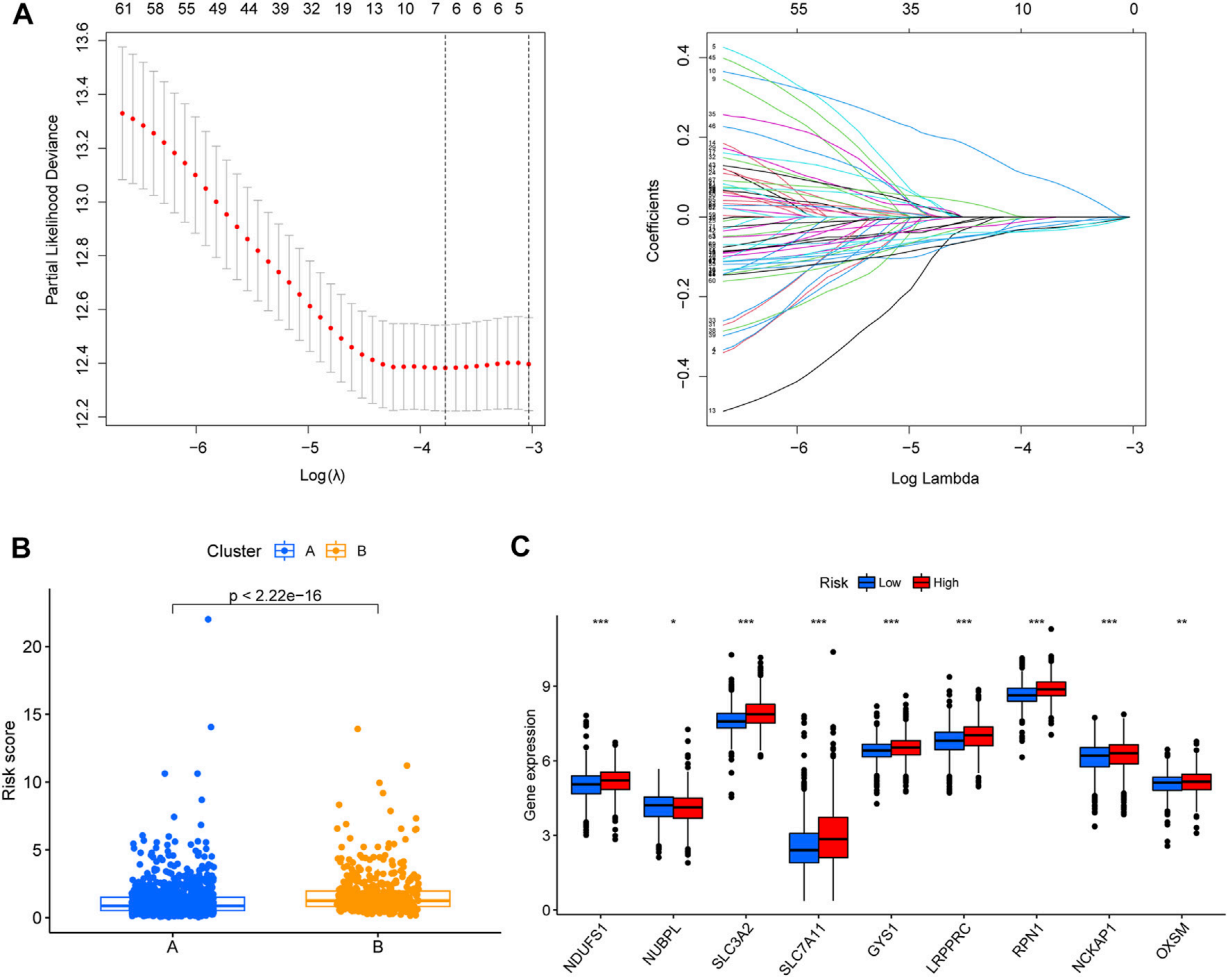
Establishment of disulfidptosis-related prognostic signature. **(A)** Lasso regression was used to establish the four-gene prognostic signature. **(B)** Box plot showing the differences in the risk score of patients between clusters **(A, B)**. **(C)** The differential gene expression analyses that were performed between low- and high-risk group. *p < 0.05, **p < 0.01, ***p < 0.001.

A total of 239 DEGs between clusters A and B, including 71 prognostic-associated disulfidptosis-DEGs were selected for univariate Cox regression analysis. A prognostic signature of four disulfidptosis-DEGs was then developed by LASSO regression analysis based on the training cohort (Figure 4A). We then verified the expression levels of four disulfidptosis-DEGs at the cellular level. KIF21A and ALOX15B were low expressed in cancer cells, APOD was high expressed in cancer cells, and ELOVL2 expression was not significantly different between cancer cells and normal cells (Supplementary Figure S2). Cluster B had a higher risk score than Cluster A (Figure 4B). Except for *NDUFA11*, the expression of nine of the DRGs differed between the high- and low-risk groups. Of these nine, *NUBPL* was expressed at low levels in the high-risk group, whereas the remaining eight genes were highly expressed in the high-risk group (Figure 4C).

### 3.6 Validation of the disulfidptosis-related prognostic signature

**FIGURE 5:**
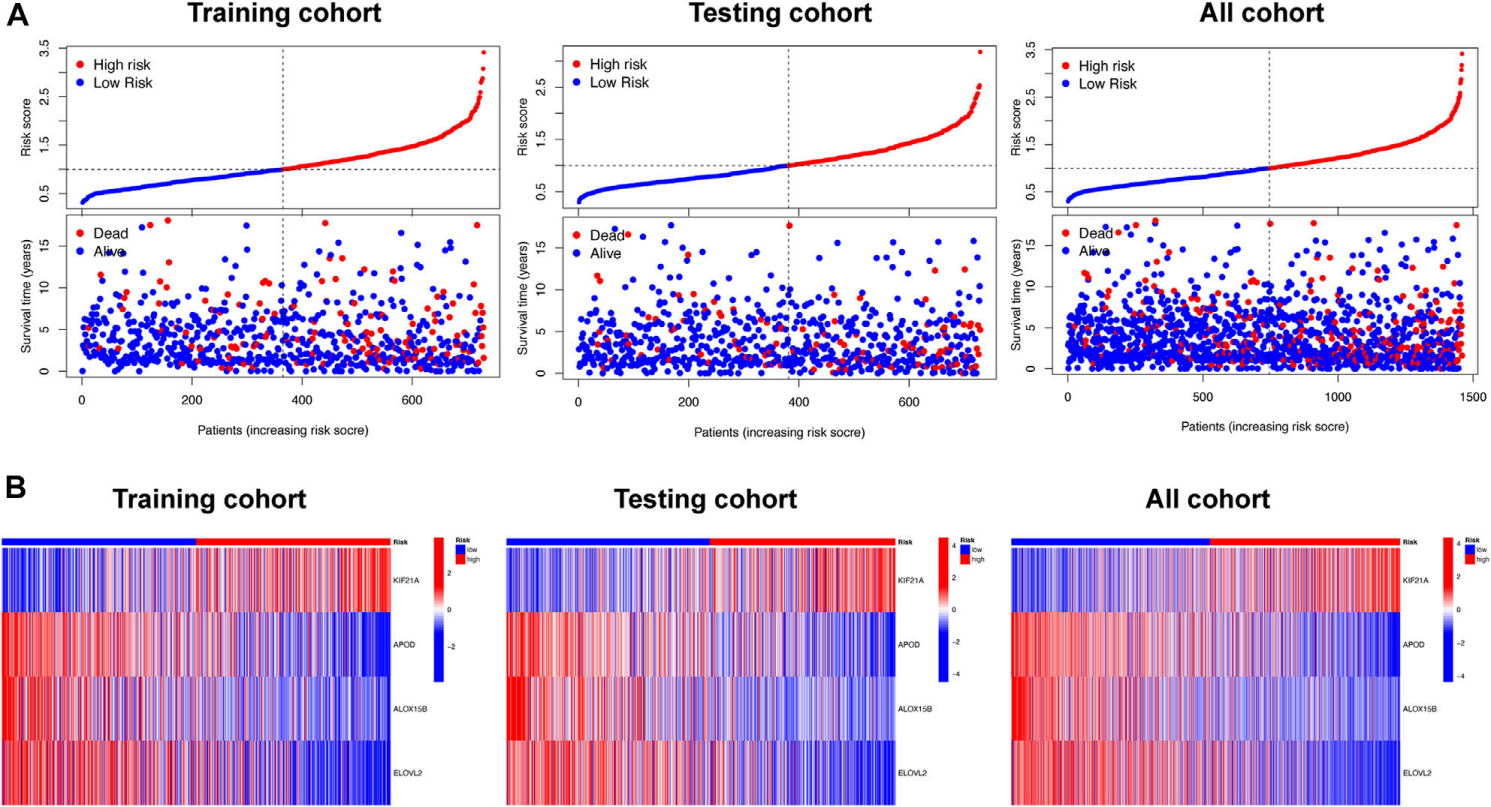
The relationship between survival status and risk score, and differential expression analysis of signature related genes, in the different risk groups. **(A)** Scatterplots showing the changes in survival statuses of BC patients as a function of increasing risk scores. **(B)** Heat map plots showing the differences between the low- and high-risk group in four signature-related genes.

The risk score of the high-risk group was higher than that of the low-risk group, and the number of deaths increased with risk score in the training and testing cohorts and all cohort (Figure 5A). A heatmap showed the differential expression of disulfidptosis-DEGs between the high- and low-risk groups (Figure 5B). Among these genes, *KIF21A* was highly expressed in the high-risk group, whereas *APOD*, *ALOX15B*, and *ELOVL2* were highly expressed in the low-risk group.

### 3.7 Evaluating the independent role of the prognostic signature and building a predictive nomogram for prognosis prediction

**FIGURE 6:**
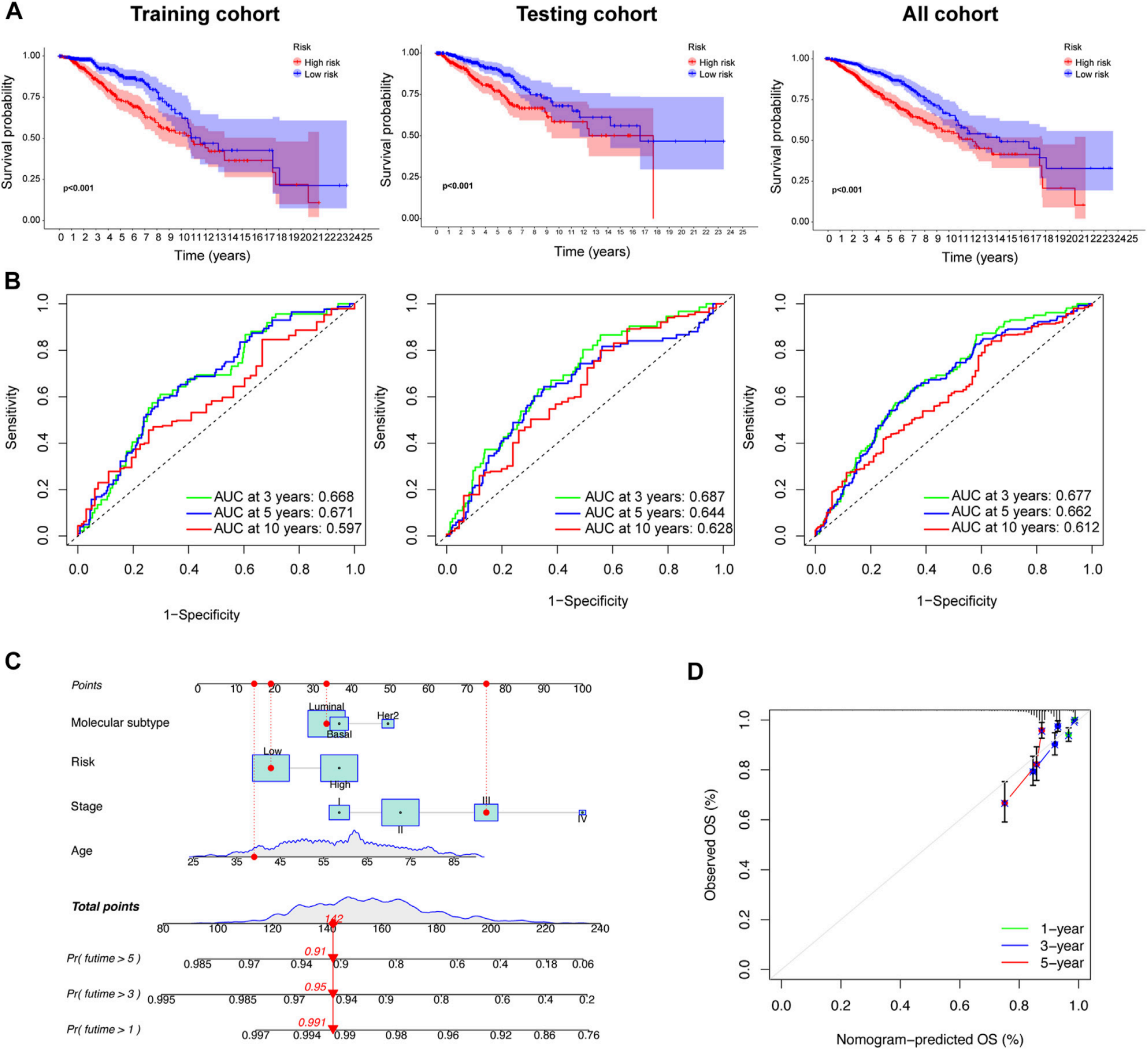
Prognostic value and reliability analyses of the prognostic signature for the training, testing, and all cohorts during development of the nomogram. **(A)** K–M survival analysis between low- and high-risk group in the three cohorts. **(B)** Receiver operating characteristic (ROC) curves were constructed, and the area under the ROC curve (AUC) were determined. **(C)** A nomogram was built based on prognostic signature and clinicopathological factors (age and disease stage). **(D)** The calibration curve showing the predictive accuracy of nomogram.

We also confirmed that the overall survival (OS) of the low-risk group was significantly longer than that of the high-risk group (*p* < 0.05; Figure 6A). We also explored the consistency of prognostic value of prognostic models across different molecular subtypes of BC. We found that in Luminal and Her2 subtypes, the PFS and DSS of high-risk group were worse than those of low-risk group. There was no difference in the prognosis of the high- and low-groups in Basel subtype, which may due to the small number of patients in the low-risk group (the number of patients with Basel subtype in the low- and the high-risk group was 14 and 175, respectively). However, we found that the 7-year PFS and DSS of the low-risk group was also significantly better than that of the high-risk group in the K-M survival curve. In general, the prognostic models had good prognostic value for different molecular subtypes of BC (Supplementary Figure S3). The AUCs of the prognostic signature suggested that the model had good predictive accuracy (Figure 6B). Nomograms are another quantitative model for predicting clinical outcomes in patients with BC. Therefore, a nomogram was developed based on the risk score and other clinical characteristics (e.g., age, disease stage and molecular subtype), so that the probability of survival at 1, 3, and 5 years for each patient with BC could be calculated (Figure 6C). The calibration charts used for internal validation of the line charts showed good agreement between the predicted OS results and actual observations (Figure 6D).

### 3.8 Analysis of immune cell infiltration, TMB, RNAss, and drug sensitivity

**FIGURE 7:**
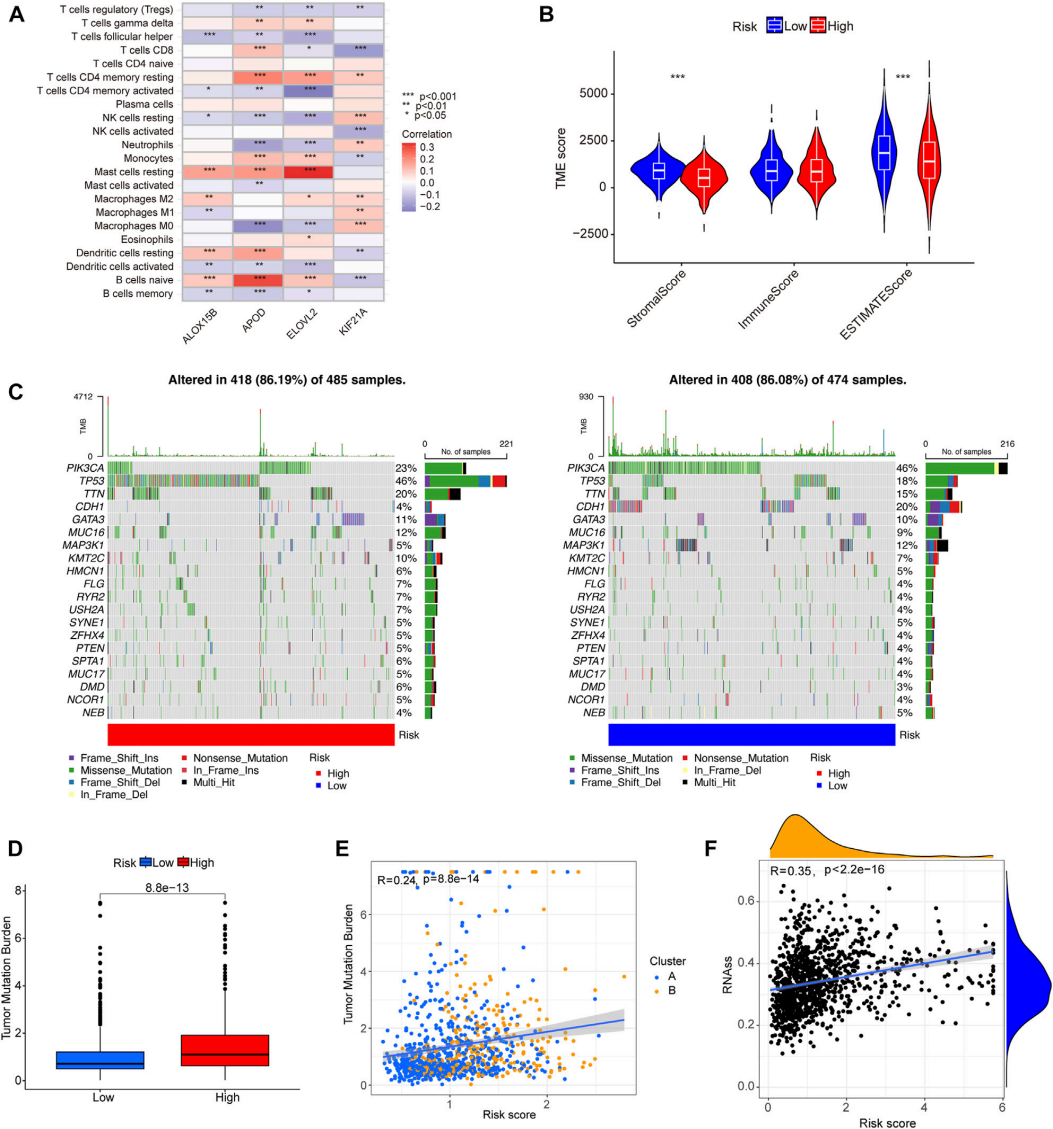
The correlation of tumor immune cell infiltration, gene mutational frequency, TMB, and RNAss with prognostic signature. **(A)** The heat map shows the correlation between four signature-related genes and level of tumor immune cell infiltration. **(B)** A violin plot showing the differences in stromal score, immune score, and estimate score between the different risk groups. **(C)** Waterfall plots showing the top 20 genes with highest gene mutational frequencies, and the types of gene mutations. **(D)** A box plot showing the difference in TMB between the low- and high-risk group. **(E)** Correlation analysis of TMB and molecular subtypes with risk score. **(F)** Correlation scatterplot showing the relationship between RNAss and risk score. *p < 0.05, **p < 0.01, ***p < 0.001.

We used the CIBERSORT algorithm to calculate the correlation between the level of infiltration of 22 immune cells and the disulfidptosis-DEGs we identified. Among these, *APOD* and naïve B cells, as well as *ELOVL2* and resting mast cells, showed significant positive correlations. *APOD* and M0 macrophages, as well as *ELOVL2* and CD4 resting memory T cells were negatively correlated (Figure 7A). We then analyzed the correlation between the content of stromal cells immune cells in the tumor microenvironment (TME), and the risk score. The low-risk group showed higher stromal and estimated scores (Figure 7B). Next, we analyzed whether there were differences in the TMB between the high- and low-risk groups. The results showed that the TMB frequency in the high-risk group was greater than in the low-risk group (Figure 7D). There was a positive correlation between TMB and risk score (Figure 7E). In BC, the TMBs of 20 genes with high mutation frequencies differed significantly between the high- and low-risk groups. For example, the mutation frequencies for *PIK3CA* were 23% and 46% in the high-and low-risk groups, respectively. *TP53* was mutated in 46% of the high-risk group and 18% of the low-risk group (Figure 7C). A positive correlation between RNAss and risk score was observed in tumor stemness analysis (Figure 7F). The results of drug sensitivity analysis showed that the sensitivity of low-risk group to cisplatin, cyclophosphamide, docetaxel, lapatinib, paclitaxel, and tamoxifen was higher than that of high-risk group, while the drug sensitivity of high-risk group to Ribociclib was higher than that of low-risk group, which could help to guide the selection of clinical treatment (Supplementary Figure S4).

## 4 Discussion

Cell energy metabolism is a necessary condition for maintaining biological development and internal environmental balance (Vander Heiden et al., 2009). Studies have shown that disulfide is closely related to energy metabolism in cancer cells. Cancer cells typically exhibit increased glucose uptake and, in the context of high SLC7A11 expression, limit NADPH production by glucose starvation or GLUT inhibition, resulting in massive accumulation of disulfide, defective oxidation-reduction reactions, and cell death (Stockwell et al., 2017; Liu et al., 2020).Disulfidptosis has recently been identified as a new type of cell death (Liu et al., 2020; Liu et al., 2023), and a new therapeutic approach for targeting and killing cancer cells. Targeting and killing of cancer cells is a new therapeutic approach. Aberrant expression of the cystine transporter solute carrier family 7 member 11 (SLC7A11; also known as xCT), the 11th member of the seventh family of solute transporters, is a cystine/glutamate anti-transporter involved in amino acid transport across the plasma membrane (Conrad et al., 2018). In 2020, Gamboi et al. found that for cells to maintain cystine at non-toxic levels, cancer cells with high expression of SLC7A11 reduced cystine to more soluble cysteine, leading to the rapid depletion of NADPH pools and abnormal accumulation of disulfides, with resultant toxic effects that led to cell death (Liu et al., 2020). We found that the expression of SLC7A11 in BC tissues was much higher than that in adjacent normal tissues. Therefore, targeting the disulfidase pathway is a promising new strategy for BC therapy.

BC is a molecular heterogeneous disease. The classical molecular subtypes of BC classify patients into Luminal, HER2 and Basel subtypes and the prognostic characteristics and drug sensitivity are different among these molecular subtypes (Holm et al., 2017). In this study, we investigated the relationship between expression patterns we built and classical molecular subtypes of BC. We found that the proportion of patients with Luminal A subtype in cluster A is significantly higher than that in cluster B, and the proportion of patients with Basel subtype in cluster B is significantly higher than that in cluster A. The epidemiological study of breast cancer reported that the prognosis of luminal A is the best among four molecular subtypes, on the contrary, the basel subtype had the worst prognosis. This is also consistent with the results in survival analysis between cluster B and cluster A in our research. Besides, the results of subgroup analysis based on the three BRCA subtypes (Luminal, Her2, Basel) indicated that the expression pattern we identified can combined with BRCA molecular subtype for better predicting and improving the prognosis of patients with luminal subtype.

At present, there are few studies on constructing prognostic models based on disulfidptosis-related gene. Recent studies have found that disulfidptosis-related gene signature has an excellent ability to identify the immune landscape of patients with bladder cancer and predict their prognosis (Zhao et al., 2023).However, little research has been conducted on DRGs in BC. Therefore, in this study, we first integrated TCGA data and the GSE86166 dataset to screen three DRGs (*NDUFS1*, *LRPPRC*, and *SLC7A11*) with differential expression and prognostic value. According to the expression pattern of DRGs, BC patients were divided into two clusters, with significant differences in OS rate and immune cell infiltration level. Indicating that these DRGs participate in TME. Subcomponent PCA was used to evaluate the prognostic value of the two groups (clusters A and B). Subsequently, four disulfidptosis-DEGs with prognostic value were identified using LASSO Cox regression analysis, and a prognostic model was constructed. In the training and validation cohort, the OS difference between the high-risk group and the low-risk group indicates that the risk score can be used as an indicator to distinguish the BC survival rate. Multivariate Cox analysis showed that risk score, age and tumor stage were considered to be independent prognostic indicators of BC. In order to better quantify 1-year, 3-year, and 5-year OS in BC patients, a nomogram combining these independent prognostic factors was developed. The results of ROC and calibration curve showed that the nomogram had significant prognostic performance. This quantitative result can be used as a complementary tool to improve prognosis assessment and personalized treatment.

The tumor microenvironment includes a variety of complex cellular components, such as immune cells, stromal cells and tumor cells (Shi et al., 2022; Srinivasan et al., 2022). Their difference in composition and expression is one of the main causes of tumor heterogeneity. Elucidating tumor immune heterogeneity will help to identify effective synergistic targets to enhance the efficacy of BC therapy. The prognosis of cluster A was better than cluster B. Cluster A showed abundant infiltration of activated B, CD8^+^ T, dendritic, natural killer cells and neutrophil. These immune cells kill tumor cells and promote immune responses and immunotherapy. In the constructed signature based on disulfidptosis-DEGs, the stromal and estimated scores of the low-risk group were higher than those of the high-risk group, and the immune score was also higher in the low-risk group than in the high-risk group, although the difference was not statistically significant. These findings suggest that disulfidptosis is associated with TME, and can be used to guide targeted immunotherapy.

Disulfidptosis is a novel type of cell death, and this study established a prognostic model based on disulfidptosis-DEGs for the first time. Our study adds to the understanding of the molecular biology of DRGs in BC. TCGA and GEO data were integrated to expand the sample size and improve the accuracy of the results. However, our study also had several limitations. First, this study mainly used the TCGA and GEO databases for analysis, and thus lacked real-world research, which urgently needs to be used for full verification of our results in the future. Second, the regulatory mechanism of DRGs in BC immune infiltration remains unclear, and further functional verification at tissue, cell and animal level is needed in the future. Finally, further research is needed to determine whether the model can be used to predict resistance to therapeutic agents in clinical practice.

## 5 Conclusion

We used consensus clustering to identify two disulfidptosis-molecular subtypes in breast cancer with different OS. We further constructed a prognostic signature based on disulfidptosis-DEGs that better predicted patient survival outcomes and tentatively identified the relationship between our risk model and the immune landscape. The results of our study provide useful insights into predicting the prognoses of patients with BC, and may even aid their treatment in clinical practice.

## Data Availability

The datasets presented in this study can be found in online repositories. The names of the repository/repositories and accession number(s) can be found in the article/Supplementary Material.
