# Supplementary material for: A novel disulfidptosis-associated expression pattern in breast cancer based on machine learning
Source: Front Genet. 2023 Jun 29;14:1193944. doi: 10.3389/fgene.2023.1193944 (PMC10343428; doi:10.3389/fgene.2023.1193944)
Supplement: Supplementary file 1 [file DataSheet1.docx]

Supplementary Material

A Novel Disulfidptosis-Associated Expression Pattern in Breast Cancer based on machine learning

**Zhitang Wang****^1^****^†^, Xianqiang Du^1†^, Weibin Lian^1^, Jialin Chen^1^, Chengye Hong^1^, Liangqiang Li****^1^, Debo Chen^1*^**

^1^Department of Breast, The First Hospital of Quanzhou Affiliated to Fujian Medical University, Quanzhou 362000, People's Republic of China

*** Correspondence:**Debo Chen
deboqz@163.com

† These authors contributed equally to this work.

| Primer | 5' to 3' |
| --- | --- |
| H-KIF21A-F | AGTTCAGCAGGATAAGGGCATAA |
| H-KIF21A-R | TTATTTCCTGCCCAGTCACCA |
| H-APOD-F | TGCATCCAGGCCAACTACTC |
| H-APOD-R | TACGGTGCCGATGGCATAAA |
| H-ELOVL2-F | TACCCTGGACAGCGCATCG |
| H-ELOVL2-R | AGTCCAACATGAACCACCCTC |
| H-ALOX15B-F | GCCACCCTCTCTTCAAGTCC |
| H-ALOX15B-R | TACCCCAGATCTGCATCCCA |
| GAPDH-F | GGTGTGAACCATGAGAAGTATGA |
| GAPDH-R | GAGTCCTTCCACGATACCAAAG |

**Supplementary Table 1.** The primer sequences involved in this study.

**
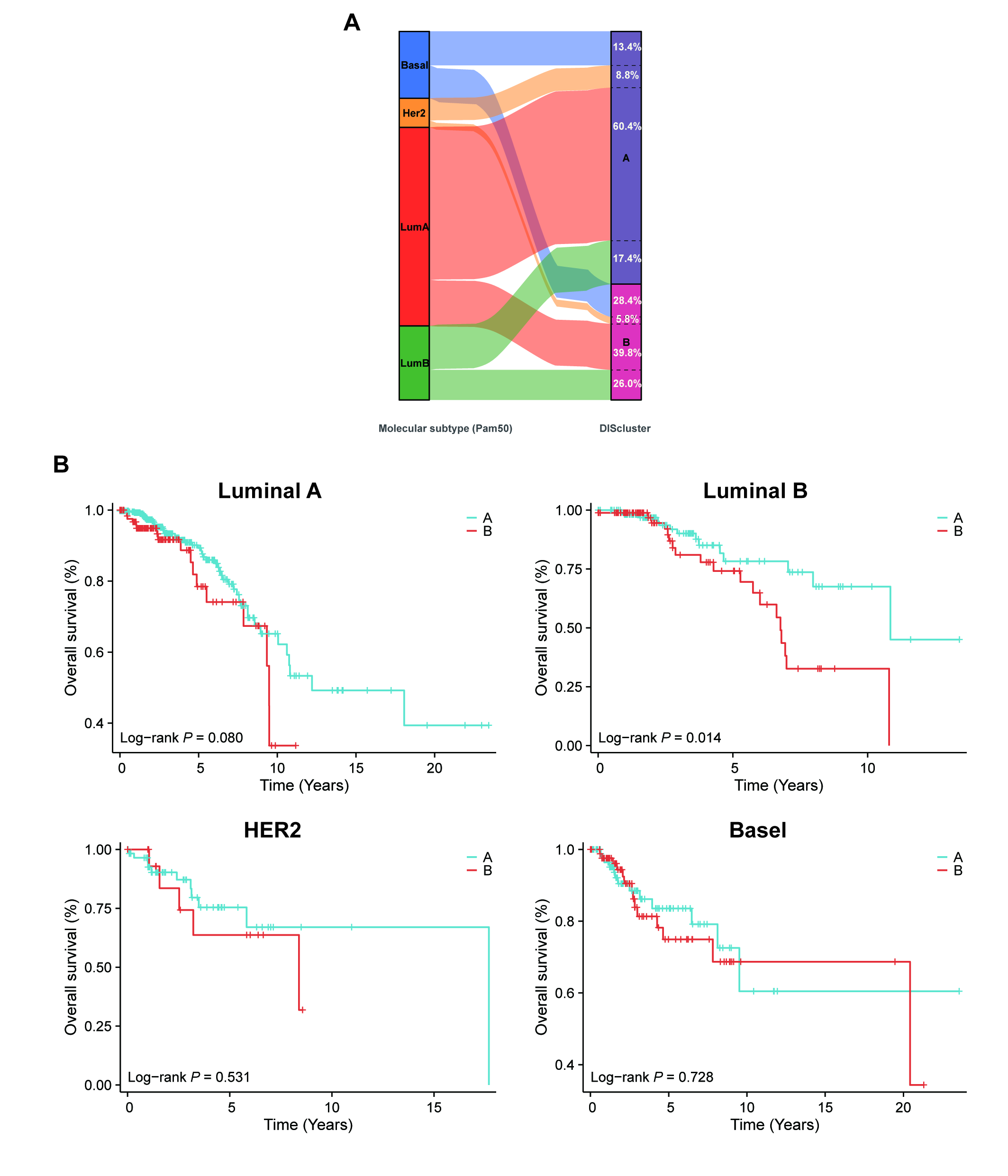
**

**Supplementary Figure S1.** (A) The Sankey diagram shown that in cluster A, patients with luminal A, luminal B, HER2 and Basel subtypes account for 60.4%, 17.4%, 8.8% and 13.4%, respectively. In cluster B, luminal A, luminal B, HER2 and Basel subtypes accounted for 39.8%, 26.0%, 5.8% and 28.4%, respectively. (B)K-M survival curve shown the difference in overall survival between two clusters.

**
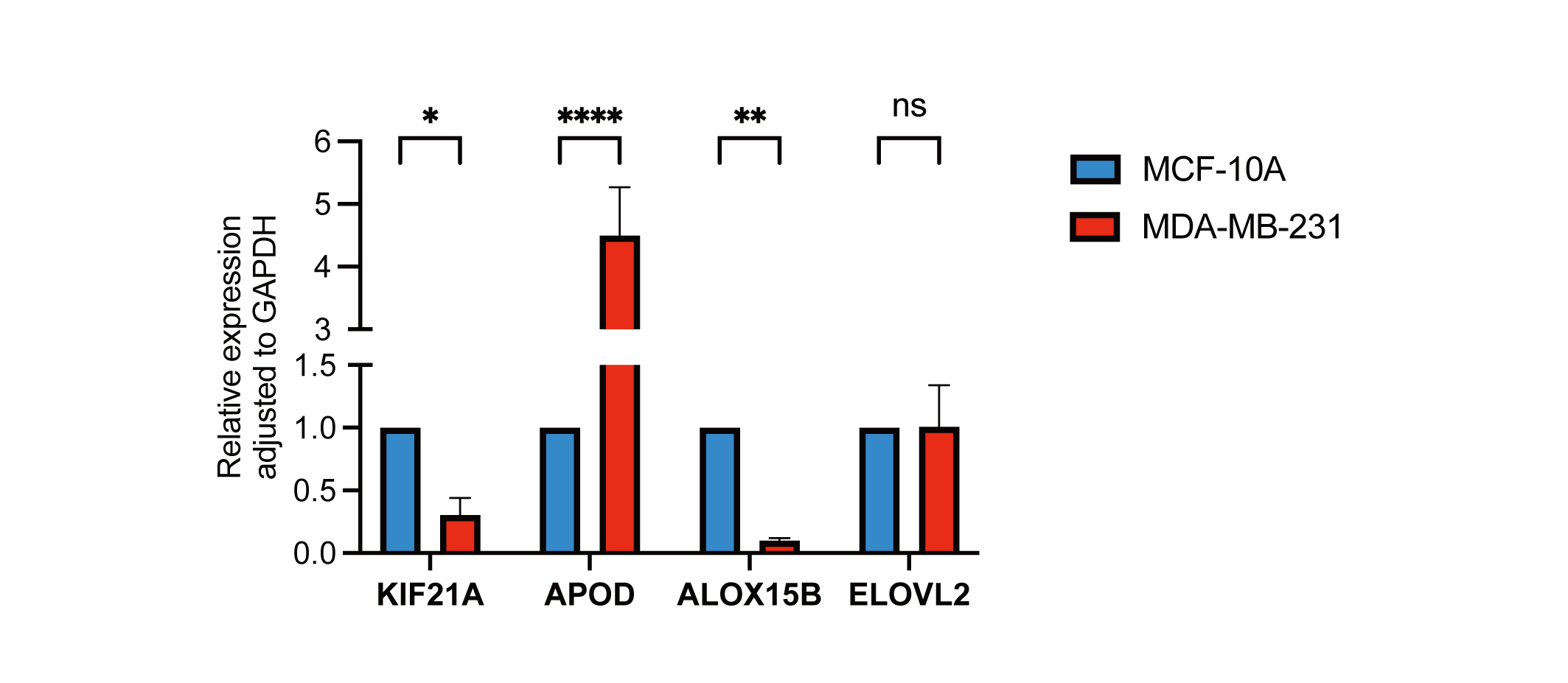
**

**Supplementary Figure S2.** Expression levels of four disulfidoptosis-DEGs in Cellular level. **p* < 0.05, ***p* < 0.01, ****p* < 0.001, ns, no significance.

**
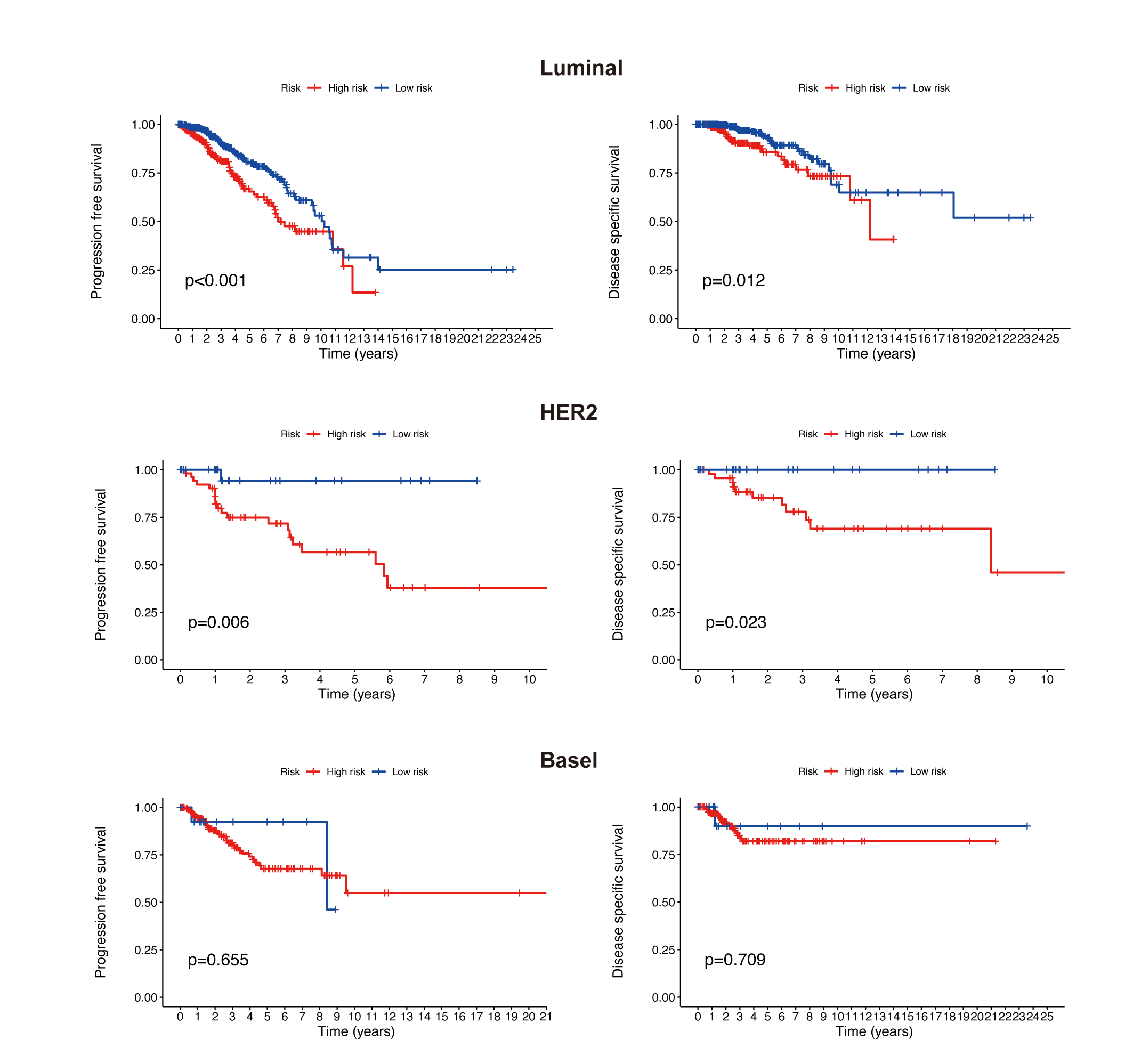
**

**Supplementary Figure S3.** The K-M survival curves shown the difference in prognosis between the low- and high-risk groups.

**
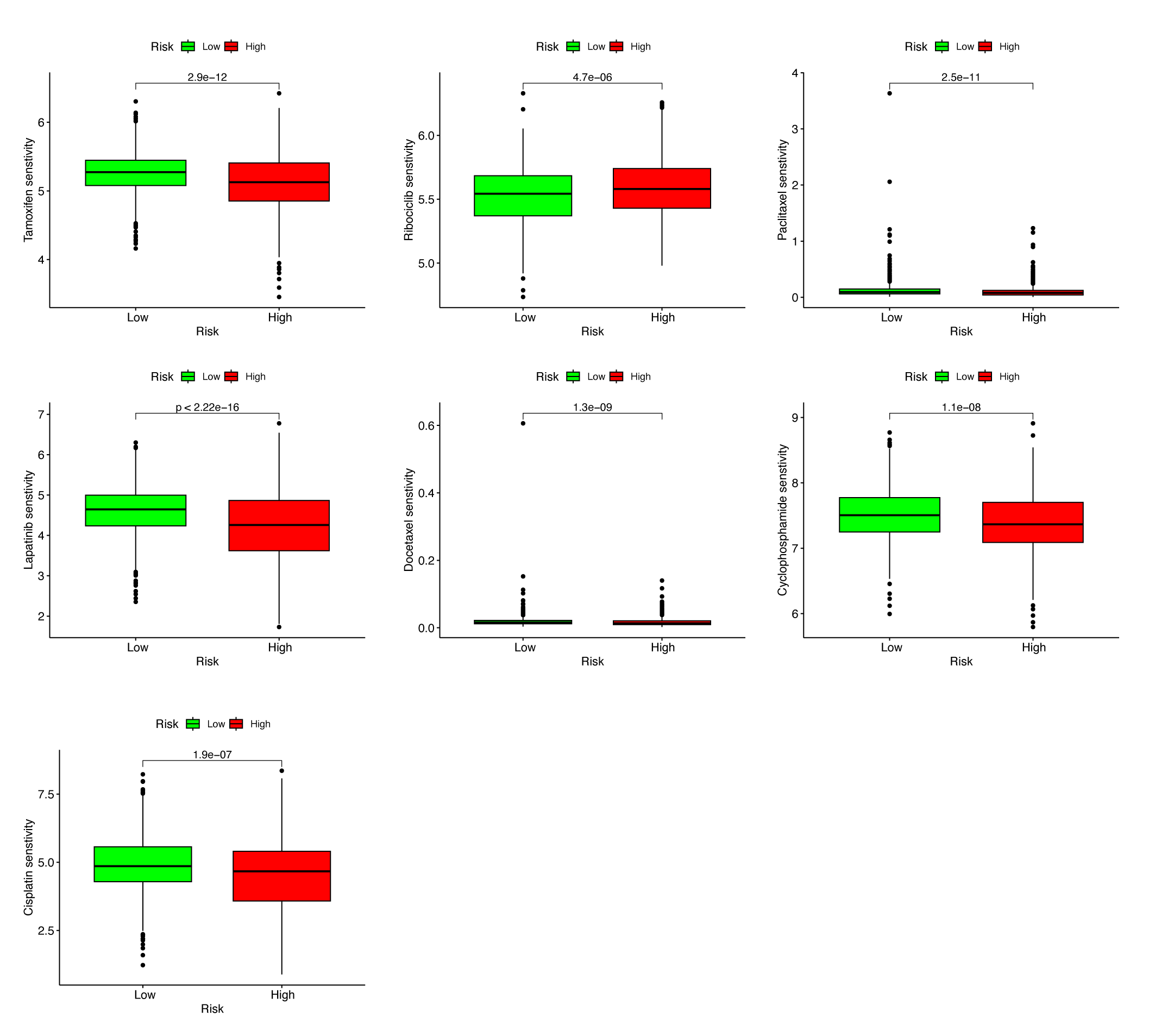
**

**Supplementary Figure S4.** The drug sensitivity analyses based on the "oncoPredict" R package to compare the differences in drug sensitivity of drugs commonly used in the treatment of BC patients between high- and low-risk groups.
